# Intraoperative visualization

**DOI:** 10.3171/2021.10.FOCVID21216

**Published:** 2022-01-01

**Authors:** Constantinos Hadjipanayis, Steven N. Kalkanis, John Y. K. Lee, Nader Sanai

**Affiliations:** 1 Department of Neurosurgery, Icahn School of Medicine at Mount Sinai, Mount Sinai Health System, New York, New York;; 2 Department of Neurosurgery, Henry Ford Health System, Detroit, Michigan;; 3 Department of Neurosurgery, University of Pennsylvania School of Medicine, Philadelphia, Pennsylvania; and; 4 Department of Neurosurgery, Ivy Brain Tumor Research Center, Barrow Neurological Institute, Phoenix, Arizona

## INTRODUCTION

Intraoperative visualization has been the driving force for neurosurgery advancement since the genesis of our field over 100 years ago. The ability to localize and delineate normal from abnormal structures has permitted more safe and effective surgeries. More than 5 decades has passed since the introduction of the conventional microscope in neurosurgery, which is used for proper illumination and magnification. Over 3 decades has now passed since the introduction of frameless stereotactic navigation, which has permitted localization and image-guided approaches in neurosurgery. The operative microscope and neuronavigation led to scientific revolutions, or paradigm shifts, in neurosurgery when they were initially described. We are now at the crossroads of intraoperative visualization again with new technologies that can enhance our ability to localize and delineate structures more so than ever before in our history of neurosurgery. These disruptive technologies represent new paradigm shifts in how we utilize technology to enhance our abilities to perform our surgeries more safely and effectively. Newer intraoperative visualization technologies such as the endoscope, exoscope, fluorescence-guided surgery, augmented reality, tractography, and heads-up display may permit better visualization and delineation of structures and lesions in the central nervous system during neurosurgery. This issue of *Neurosurgical Focus: Video* on intraoperative visualization provides the latest intraoperative technologies in video case format. We thank all the authors for their timely submissions and moving neurosurgery forward with their innovative applications of intraoperative visualization technologies.

## Disclosures

Dr. Hadjipanayis is a consultant for Synaptive Medical, NX Development Corporation (NXDC), Stryker Corporation, and Hemerion. He receives financial compensation as a consultant and lecturer for Synaptive (manufacturer of the Synaptive MODUS V device). NXDC, a privately held company, markets Gleolan (5-aminolevulinic acid hydrochloride [5-ALA]), and Dr. Hadjipanayis receives royalty payments for the sale of Gleolan. Dr. Kalkanis is a consultant for Synaptive and Arbor Pharma. Dr. Lee is a consultant for VisionSense.

